# Is plaque regrowth inhibited by dentifrice? A systematic review and meta‐analysis with trial sequential analysis

**DOI:** 10.1111/idh.12364

**Published:** 2018-09-27

**Authors:** Cees Valkenburg, Fridus Van der Weijden, Dagmar Else Slot

**Affiliations:** ^1^ General Dentist and Clinical Epidemiologist Hoevelaken The Netherlands; ^2^ Department of Periodontology Academic Centre for Dentistry, Amsterdam (ACTA) University of Amsterdam and Vrije Universiteit Amsterdam Amsterdam The Netherlands

**Keywords:** dentifrice, oral hygiene, plaque, regrowth, systematic review, toothbrushing, toothpaste

## Abstract

**Objectives:**

The aim of this systematic review was to establish in studies with human participants the effect of a regular fluoride dentifrice compared to water or saline on dental plaque inhibition.

**Methods:**

MEDLINE‐PubMed, Cochrane‐CENTRAL, EMBASE and other electronic databases were searched, up to April 2018. The inclusion criteria were controlled clinical trials among participants aged ≥18 years with good general health. Papers that evaluated the effect of dentifrice slurry compared with water or saline on plaque regrowth during a 4‐day nonbrushing period were included. Data were extracted from the eligible studies, the risk of bias was assessed, and a meta‐analysis was performed where feasible.

**Result:**

The search retrieved eight eligible publications including 25 comparisons. The estimated potential risk of bias was low for all studies. Based on three different indices, overall plaque regrowth was significantly (*P *<* *0.01) inhibited for 0.25 or more by the use of a dentifrice slurry as compared to water. All subanalysis on specific dentifrice ingredients and the overall descriptive analysis supported these findings.

**Conclusion:**

The results of this review demonstrate moderate‐quality evidence for a weak inhibitory effect on plaque regrowth in favour of the use of a dentifrice intended for daily use.

## 1 INTRODUCTION

Good oral hygiene results in the reduction in plaque, caries and gingivitis.^1^ Toothbrushing is effective in reducing levels of dental plaque.^2^ It is generally accepted that dentifrice should be used in combination with a toothbrush,^3^ although plaque reduction can be achieved without.^4^, ^5^ Adding dentifrice to a toothbrush does not appear to improve the shear force that is exerted on the plaque biofilm through the scrubbing effect of the toothbrush filaments.^6^ But this finding does not imply that brushing without a dentifrice should be recommended primarily due to the lack of fluoride to prevent caries.^7^

As the available scientific literature suggests that dentifrices do not improve the mechanical action of brushing on plaque removal,^8^ a further aspect of interest is whether dentifrice reduces plaque regrowth. Many plaque growth studies have reported a reduction in regrowth of plaque between brushings.^9^, ^10^, ^11^, ^12^ However, evaluating this influence was complicated by the ever‐present variable of the participants’ toothbrushing efficacy.^11^, ^13^ The mechanical action of the toothbrush during a test period obscures the antiplaque effect of the dentifrice by itself.^13^ Also, the Hawthorne effect, whereby oral hygiene practices are improved irrespective of the test product, can easily occur in oral hygiene study designs. To some incalculable degree, it could mask the true adjunctive effect of the dentifrice,^14^ making it impossible to determine whether the reduction in plaque regrowth results from very efficient brushing or from a chemical antiplaque effect of the dentifrice.^15^ One proposed alternative is to assess the effects of dentifrice ingredients on plaque regrowth independently of those of mechanical cleaning effect of a toothbrush by delivering the dentifrice formulation as a slurry in mouthwash form.^15^, ^16^ To obtain a slurry, the dentifrices are mixed with water so that simple rinsing reproduces the quantity of active substance present in the oral cavity during normal toothbrushing, without the mechanical cleaning effect of toothbrushing.^17^ A suitable research model for investigating whether dentifrice can play a role as plaque‐reducing agent seems to be the 4‐day nonbrushing model developed by Addy et al^15^ This design has been used extensively and allows the chemotherapeutic activity of dentifrice products on dental plaque to be rapidly determined.^18^

The objective of this systematic review (SR) was therefore to systematically and critically appraise the literature on 4‐day nonbrushing models that compared the efficacy on plaque regrowth of a dentifrice for daily use with that of water or saline only.

## 2 MATERIALS AND METHODS

This SR was prepared and described in accordance with the Cochrane Handbook for Systematic Reviews of Interventions^19^ and the guidelines of Transparent Reporting of Systematic Reviews and Meta‐analyses (PRISMA statement).^20^ The protocol that details the review method was developed “a priori*”* following an initial discussion among the members of the research team.

### 2.1 Focused question

What is the efficacy of a regular dentifrice intended for daily use on regrowth of dental plaque used as a slurry in comparison with that of water or (sterile) saline in healthy adults?

### 2.2 Search strategy

A structured search strategy was designed to retrieve all relevant studies. The National Library of Medicine, Washington, D.C. (MEDLINE‐PubMed), the Cochrane Central Register of Controlled Trials (CENTRAL) and EMBASE (Excerpta Medica Database by Elsevier) were searched from initiation to April 2018 for appropriate papers that answered the focused question. The reference lists of the included studies were hand‐searched to identify additional potentially relevant studies. For details regarding the search terms used, see Table 1.

**Table 1:** Search terms used for PubMed‐MEDLINE, Cochrane‐CENTRAL and EMBASE. The search strategy was customized according to the database being searched

The following strategy was used in the search:
{[<intervention>] AND [<outcome>]}
{[<intervention: toothpaste>
([MeSH terms/all subheadings] toothpastes)
OR
([text words] toothpaste OR dentifrice OR toothpastes OR dentifrices)]
AND
[<outcome: dental plaque>
([*MeSH terms/all subheadings*] dental plaque OR dental Plaque Index OR dental deposits)
OR
([text words] plaque OR plaque removal OR Plaque Index OR dental plaque OR interdental plaque OR interproximal plaque)
AND
([text words] overnight OR growth OR regrowth)]}

### 2.3 Screening and selection

The titles and abstracts of the studies obtained from the searches were screened independently by two reviewers (C.V. and D.E.S.) to select studies that potentially met the inclusion criteria. No language restrictions were imposed. Based on the title and abstract, the full‐text versions of potentially relevant papers were obtained. These papers were categorized (by C.V. and D.E.S.) as definitely eligible, definitely not eligible or questionable. Disagreements concerning eligibility were resolved by consensus, and if disagreement persisted, the decision was resolved through arbitration by a third reviewer (G.A.W.). The papers that fulfilled all the inclusion criteria were processed for data extraction.

The included studies were considered to meet the following criteria: (a) the study design was either a randomized controlled clinical trial (RCT) or a controlled clinical trial (CCT). (b) The studies were conducted with humans, who were not institutionalized and were 18 years of age or older. (c) The studies only included participants who were in good general health (no systemic disorders) and were without orthodontic appliances and/or removable prostheses. (d) The studies used a nonbrushing 4‐day plaque regrowth model. (e) The intervention was a slurry from a regular dentifrice, and the comparator was water or saline. (f) The studies evaluated any plaque scores. (g) The publications were available as full reports.

### 2.4 Assessment of heterogeneity

The following factors were used to evaluate the clinical and methodological heterogeneity of the outcomes of the different studies: study design, subject characteristics, study group details, side effects and industry funding.

### 2.5 Study quality and risk of bias assessment

Two reviewers (C.V. and D.E.S.) independently scored the individual methodological qualities of the included studies using the checklist presented in Appendix S2. In short, a study was classified as having a low risk of bias when random allocation, defined inclusion/exclusion criteria, blinding to patient and examiner, balanced experimental groups, identical treatment between groups (except for the intervention) and reporting of a follow‐up were present. Studies that met six of these seven criteria were considered to have a potential moderate risk of bias. If two or more of these seven criteria were absent, the study was considered to have a high risk of bias as proposed by Van der Weijden et al^21^ and described in detail by Keukenmeester et al^22^

### 2.6 Data extraction

The characteristics of the population, intervention, comparison and outcomes were extracted independently from all studies by two reviewers (C.V. and D.E.S.) using a specially designed data‐ extraction form. Data applied in crossover design studies were assessed as those from parallel designs. Means and standard deviations (SDs) were extracted. Disagreement between the reviewers was resolved through discussion and consensus. If a disagreement persisted, the judgement of a third reviewer (G.A.W.) was decisive. Some studies provided standard errors (SEs) of the means. Where possible, the authors calculated standard deviation based on the sample size (SE = SD/√N) and transformed logarithmic value back to the raw scale.^23^ Where applicable, inches were converted to centimetres and totals to averages. For those papers that provided insufficient data to be included in the analysis, the first or corresponding author was contacted to request additional data.

### 2.7 Data analysis

As a summary, a descriptive data presentation was used for all studies. Subsequently, where feasible, a meta‐analysis (MA) was performed. Subgroup analyses were conducted according to different chemotherapeutic ingredients with a minimum of two included experiments. For studies that had multiple treatment arms and for which data from the control group were compared with more than one other group, the number of participants (n) in the control group was divided by the number of comparisons if the number of participants in the control group would not be smaller than seven. When appropriate and desirable, multiple treatment arms would be combined into a single group. The data are presented, and the modifications of the original indices 15, 24, 25, 26, 27 are provided. The difference of means (DiffM) between the test and control groups was calculated using a “random‐effects” model with an “inverse variance” method as proposed by DerSimonian and Laird.^28^ For MA with more than two comparisons, 95% predictive intervals were calculated to quantify treatment effects in a future clinical setting.^29^ Heterogeneity was tested using the chi‐square test and the *I* ^2^ statistic with 95% confidence intervals around *I* ^2^.^19^, ^30^ If possible, the formal detection testing for potential publication bias was used as proposed by Egger et al^31^ and Sterne et al^32^ Post hoc analysis was conducted on study design. Computations for the MA were performed using R (https://www.r-project.org) with the packages meta 33, 34 and metafor.^35^ Trial sequential analysis was applied to reduce the risk of type Ι error. The required information size (RIS) and the trial sequential monitoring boundaries (TSMB) for benefit or futility were calculated. The RIS was calculated based on a type Ι error risk of α = 5% and a type ΙΙ error risk of β = 0.20, with a statistical test power of 80%. RIS was accounted for heterogeneity and multiple comparisons. The Lan‐DeMets version^36^ of the O'Brien‐Fleming function^37^ was used for calculating the TSMBs. TSA software version 0.9.5.10 Beta (Copenhagen Trial Unit, Copenhagen, Denmark) was used.^38^, ^39^, ^40^, ^41^, ^42^

### 2.8 Grading the “body of evidence”

The Grading of Recommendations Assessment, Development and Evaluation (GRADE) system was used as proposed by the GRADE‐working group 43 to appraise the evidence emerging from this review. Two reviewers (G.A.W. and D.E.S.) rated the quality of the evidence, and any disagreement between the two reviewers was resolved after additional discussion with a third reviewer (C.V.).

## 3 RESULTS

### 3.1 Search and selection results

**Figure 1:**
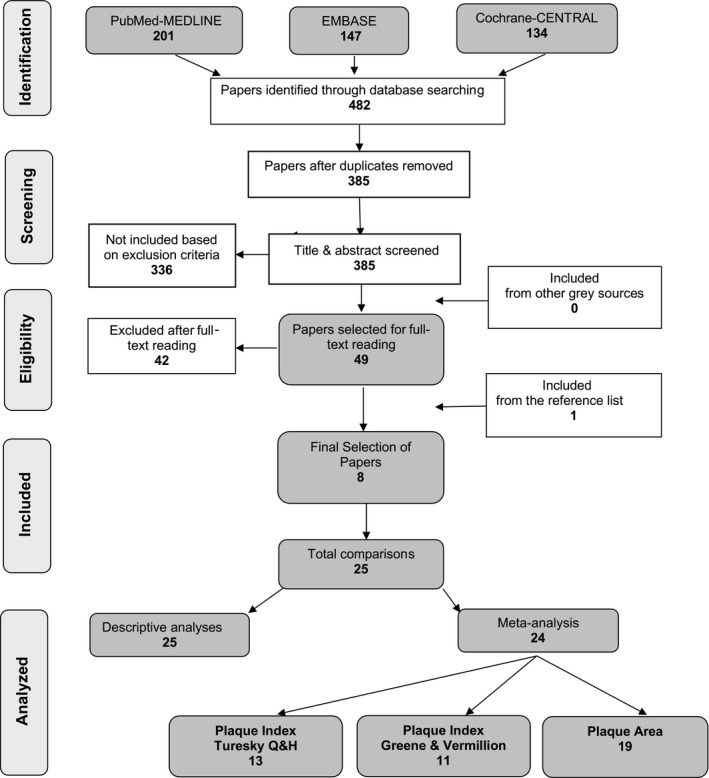
Search and selection results

The search of the MEDLINE‐PubMed, Cochrane‐CENTRAL and EMBASE databases resulted in 195 unique papers (for details, see Figure 1). Manual searching of the reference lists of the eight selected papers provided one additional relevant paper. Altogether, nine eligible publications in which described 25 comparisons were included in this SR.

### 3.2 Study characteristics, heterogeneity and funding

The included studies exhibited moderate heterogeneity. Information regarding the study characteristics is provided in detail in Appendix S1. All the studies had a crossover design. The washout periods between treatments herein varied from 3 to 10 days.^44^, ^45^ The dentifrices used in the studies exhibited a large variation in brands, compositions and concentrations of the ingredients. All but one study 15 provided the name of the dentifrice brands. Two noncommercial dentifrices were used in one study.^46^

Rinsing under supervision was performed in four of the studies.^44^, ^45^, ^47^, ^48^ The rinsing time was 1 minute in all but one study.^44^ In this study, 30 seconds of rinsing with a dentifrice slurry was preceded by rinsing 30 seconds with water. No side effects were reported in the included studies besides burning sensations. Binney et al 47 reported of five participants with transient mouth burning during the trial, although none were of a severity that required the trial randomization to be broken. All the participants in the study of Arweiler et al^17^ observed a slight burning sensation. One study acknowledged that the study was independently performed by the authors and was not supported by any research grant or commercial organization.^16^ Three studies did not mention support or assistance from a commercial partner.^15^, ^17^, ^45^ Two studies acknowledged support and assistance from industry 46 (Colgate‐Palmolive Company),^48^ (Procter and Gamble). None of the studies included a disclosure statement for conflict of (financial) interests. However, several authors in the studies mentioned affiliations with industry 44, 47, 48 (Procter and Gamble).

### 3.3 Methodological quality and risk of bias assessment

To estimate the potential risk of bias, the methodological qualities of the included studies were used, as assessed in the checklist presented in Appendix S2. The procedures for allocation concealment were not described in any of the selected studies. Because the current study was focused on the adjunctive use of a dentifrice, blinding to the intervention was not applicable. All studies provided a professional prophylaxis to remove all plaque, stains and calculus at baseline. Two studies performed a sample size as well as power calculation and mentioned an intention‐to‐treat analysis.^44^, ^45^ Four of the studies did not provide information about examiner calibration.^15^, ^16^, ^45^, ^46^ Based on a summary of the proposed criteria, the estimated potential risk of bias was low for all studies.

### 3.4 Study outcomes/results

Appendix S3 presents the results of the data extraction per index used. The outcomes by and relative to the Plaque Index as well as the plaque area score are presented in the current study.

#### 3.4.1 Descriptive analysis

Table 2 provides a summary of the differences reported to be significant between rinsing with a dentifrice slurry as compared to rinsing with water or (sterile) saline alone, as reported by the original authors. Twenty‐two comparisons out of 25 demonstrated a significant difference between interventions in favour of the use of a dentifrice slurry on the Plaque Index used. Only three comparisons indicated no significant difference.^15^, ^16^, ^44^ All but one 16 of the 21 comparisons also exhibited a significant difference in favour of the use of a dentifrice slurry according to the Plaque Area Index. This was irrespective of the specific dentifrice ingredient or when a comparison as saline or water used.

**Table 2:** A descriptive summary of the statistical significance of individual study outcomes related to the effect of rinsing with a dentifrice slurry or with water on dental plaque in a 4‐day plaque regrowth design

Study #	Intervention rinsing dentifrice slurry with	Plaque Index		Plaque area	Comparison rinsing with
Addy et al. (1983) 15	Sodium Fluoride	=	(G&V)	>	Water
Addy et al. (1990) 46	Sodium Fluoride	>	(G&V)	>	Saline
Binney et al. (1995) 47	Sodium Fluoride	>	(TQ&H)	>	Saline
Binney et al. (1996) 48	Sodium Fluoride	>	(TQ&H)	>	Water
Binney et al. (1996) 48	Sodium Fluoride	>	(TQ&H)	>	Water
Addy et al. (1983) 15	Monofluorophosphate	>	(G&V)	>	Water
Binney et al. (1992) 16	Monofluorophosphate	=	(TQ&H)	=	Saline
Binney et al. (1996) 48	Monofluorophosphate	>	(TQ&H)	>	Water
Owens et al. (1997) 44	Monofluorophosphate	=	(TQ&H)	□	Water
Addy et al. (1983) 15	Monofluorophosphate	>	(G&V)	>	Water
Binney et al. (1996) 48	Monofluorophosphate	>	(TQ&H)	>	Water
Addy et al. (1983) 15	Monofluorophosphate	>	(G&V)	>	Water
Addy et al. (1983) 15	Stannous Fluoride	>	(G&V)	>	Water
Addy et al. (1990) 15	Stannous Fluoride	>	(G&V)	>	Saline
Binney et al. (1997) 45	Stannous Fluoride	>	(TQ&H)	□	Water
Addy et al. (1990) 46	Stannous Fluoride	>	(G&V)	>	Saline
Addy et al. (1990) 46	Stannous Fluoride	>	(G&V)	>	Saline
Addy et al. (1990) 46	Triclosan	>	(G&V)	>	Saline
Binney et al. (1995) 47	Triclosan	>	(TQ&H)	>	Saline
Binney et al. (1996) 48	Triclosan	>	(TQ&H)	>	Water
Arweiler et al. (2002) 17	Triclosan	>	(TQ&H)	>	Water
Addy et al. (1990) 46	Triclosan	>	(G&V)	>	Saline
Binney et al. (1997) 45	Triclosan	>	(TQ&H)	□	Water
Binney et al. (1997) 45	Triclosan	>	(TQ&H)	□	Water
Arweiler et al. (2002) 17	Baking Soda	>	(TQ&H)	>	Water
Summary		= 3 x > 22 x		= 1 x > 20 x	

>, significant difference in favour of the intervention (rinse with dentifrice slurry); <, significant difference in favour of the control group (water or saline); =, no significant difference; □, no data available; BS, baking soda; Parodontax with fluoride; G&V, Greene & Vermillion Plaque Index^24^; MFP, sodium monofluorophosphate; MFP+NaF, sodium monofluorophosphate and sodium fluoride; MFP‐Zn, sodium monofluorophosphate with zinc citrate; NaF, sodium fluoride; NaF‐pyro: sodium fluoride with pyrophosphate; SnF, stannous fluoride; SnF gel, stannous fluoride gel; SnF‐SnCl, stannous fluoride with stannous chloride; Tcs, triclosan; Tcs‐co, triclosan with copolymer; Tcs‐Zn, triclosan with zinc citrate; TQ&H, Quigley and Hein (Q&H) Plaque Index^25^ by Turesky et al.^26^

#### 3.4.2 Meta‐analysis

All studies except one^44^ provided information on sample size, outcomes and standard errors/deviations. No additional data were obtained after contacting the authors. A random meta‐analysis could be performed, but the studies were separately analysed based on the index used. Subgroup analysis was performed by dentifrice ingredient. A total of 24 comparisons from eight papers involving 98 patients and 329 experiments could be included. Table 3 presents the outcomes.

**Table 3:** Overview of the data extracted from the meta‐analysis and the trial sequential analysis (TSA) on the experiments in the Plaque Index scores separated per main reported ingredient

Source					Outcomes				Heterogeneity		Trial sequential analysis (TSA)				Details see Appendix^c^
Index	Dentifrice slurry	# Studies	# exp in MA	Model	DiffM	95% CI	*p*‐value^b^	95% Prediction interval	*I* ^2^ [95% CI]	*P*‐value^b^	# participants in exp	Heterogeneity‐adjusted information size (TSA)	Maximum additional participants required	Statistical evidence (TSA)	
**TQ&H** d **Overall**		5	13	Random	−0.30	−0.38; −0.22	<0.01	[−0.58; −0.02]	87% [79%;92%]	<0.01	377	338	0	Conclusive	S4, S7‐1
Sodium Fluoride		2	3	Random^a^	−0.30	−0.33; −0.26	<0.01	[−0.52; −0.07]	0% [0%;89%]	0.38	104	338	234	Inconclusive	S4
Monofluorophosphate		2	3	Random	−0.14	−0.23; −0.04	<0.01	[−1.10; 0.82]	49% [0%;85%]	0.14	104	1007	903	Inconclusive	S4
Triclosan		4	5	Random	−0.44	−0.57; −0.32	<0.01	[−0.82; −0.06]	70% [23%;88%]	0.01	130	338	208	Inconclusive	S4
**Greene & Vermillion** e **Overall**		2	11	Random	−0.25	−0.32; −0.18	<0.01	[−0.34; −0.17]	0% [0%;0%]	1.00	280	51	0	Conclusive	S5, S7‐2
Sodium Fluoride		2	2	Random^a^	−0.24	−0.41; −0.07	<0.01	[NA]	0% [NA]	0.55	50	50	0	Conclusive	S5
Monofluorophosphate		1	3	Random^a^	−0.24	−0.35; −0.12	<0.01	[−0.98; 0.50]	0% [0%;23%]	0.87	60	28	0	Conclusive	S5
Stannous Fluoride		2	4	Random^a^	−0.26	−0.40; −0.13	<0.01	[−0.56; 0.04]	0% [0%;0%]	0.97	110	61	0	Conclusive	S5
Triclosan		1	2	Random^a^	−0.30	−0.56; −0.05	0.02	[NA]	0% [NA]	0.91	60	91	31	Inconclusive	S5
**Plaque Area** f **Overall**		5	19	Random	−0.30	−0.38; −0.23	<0.01	[−0.51; −0.09]	64% [41%;78%]	<0.01	558	82	NA	Inconclusive	S6, S7‐3
Sodium Fluoride		4	5	Random^a^	−0.35	−0.39; −0.31	<0.01	[−0.41; −0.29]	0% [0%;65%]	0.67	154	82	0	Conclusive	S6
Monofluorophosphate		3	6	Random	−0.20	−0.31; −0.08	<0.01	[−0.44; 0.05]	20% [0%;65%]	0.28	164	112	NA	Inconclusive	S6
Stannous Fluoride		2	4	Random^a^	−0.06	−0.35;0.23	0.69	[−0.69; 0.57]	0% [0%;0%]	1.00	110	112	NA	Inconclusive	S6
Triclosan		3	4	Random^a^	−0.47	−0.51; −0.43	<0.01	[−0.56;−0.38]	0% [0%;77%]	0.57	130	112	0	Conclusive	S6

CI, Confidence interval; NA, Not applicable.

a Same values with fixed model.

b The number of decimals to which the annotations have been rounded off is 2.

c Forest and funnel plots.

d Quigley and Hein (Q&H) Plaque Index^25^ by Turesky et al.^26^

e Greene and Vermillion Plaque Index.^24^

f Plaque Area Index by Addy et al.^15^.

The analysis on the available data from the modification of the Quigley & Hein (Q&H) Plaque Index^25^ by Turesky et al^26^ included five studies, which resulted in 13 comparisons. End measurements did provide a significant difference of means in favour of rinsing with a dentifrice slurry (DiffM −0.30; *P *<* *0.00001; 95% CI: [−0.38; −0.22]) (Appendix S4a). For the analysis on the available data from the Plaque Index by Greene and Vermillion,^24^ two studies with 11 comparisons could be included. The end measurements also provided a significant difference (DiffM −0.25; *P *<* *0.00001; 95% CI: [−0.32; −0.18]) (Appendix S5a). Five studies with 19 comparisons were available for the analysis of the Plaque Area Index, the modification of the Shaw and Murray 27 stain index by Addy et al^15^ The end measurements also exhibited a significant result in favour of the rinsing with dentifrice slurries (DiffM −0.30; *P *<* *0.00001; 95% CI: [−0.38; −0.23]).

All but one subanalysis based on the active ingredients for all indices of interest were statistically significant in favour of the dentifrice slurry compared to saline/water (for details, see Table 3). Unexplained heterogeneity in the meta‐analyses was high for the studies assessing the Q&H by Turesky et al 26 index (*I* ^2^ = 87%; *P *= < 0.01) and the Plaque Area Index^15^ (*I* ^2^ = 64%; *P *<* *0.01) and low for studies that assessed plaque using the Greene & Vermillion 24 index (*I* ^2 ^= 0%; *P *=* *1.00).

Although <10 publications were included, the meta‐analysis was based on 24 comparisons with a minimum of 11 experiments per index of interest. Therefore, a funnel plot was constructed.^49^, ^50^, ^51^

The funnel plots related to the meta‐analysis on the available data for the different indices of interest, presented in Appendices S4b, S5b and S6b indicate that publication bias is possible.

Three post hoc sensitivity analysis of the crossover trials was performed in order to confirm the robustness of the results of the MA.^52^ A within‐patient correlation of 0.5 was assumed because information of the required matched outcome data was not available.^19^, ^53^ See Appendix S8 for the results of the post hoc sensitivity analysis.

#### 3.4.3 Trial sequential analysis

Appendix S7 presents the results of the trial sequential analysis (TSA) per index used. TSA of this MA showed that the effect was conclusive and reliable and that additional data are unlikely to affect the summary effect.^39^

### 3.5 Evidence profile

The data gathered are indirect as the model of interest is a research model for a proof of principle. However, the data are rather consistent and precise. Table 4 shows a summary of the various factors used to rate the quality of evidence and strength of recommendations according to GRADE.^43^ The strength of a recommendation based on the quality of the evidence emerging from this review is estimated to be moderate. Given that the effect is small, the direction of recommendation emerging from this SR is weak in favour of the use of a dentifrice with the intention to inhibit regrowth of dental plaque.

**Table 4:** Summary of findings table on body of the estimated evidence profile (GRADE, 2015) and appraisal of the strength of the recommendation regarding the effectiveness of dentifrice on plaque regrowth, in 4‐day nonbrushing plaque regrowth models

Determinants of quality	Plaque scores
Study design (Appendix S1)	RCTs crossover designs
# studies n = 8	25
# comparison n = 25 (Figure 1)	
Risk of bias (Appendix S2)	Low
Consistency (Table 3)	Rather consistent
Directness (Dentifrice slurry)	Indirect
Precision (Table 3)	Rather precise
Publication bias (Appendices S4b/S5b/S6b)	Possible
Magnitude of the effect	Small
Strength of the recommendation based on the body of evidence	Moderate
Direction of recommendation	Weak in favour of the use of dentifrice

## 4 DISCUSSION

Over recent decades, dentifrice formulations have been developed to deliver chemical and physical mediated benefits.^54^ Despite these efforts, a recent SR indicated that dentifrice appears not to provide an adjuvant mechanical action of toothbrushing on the instant removal of plaque.^8^ Traditionally, dentifrices have played an important role in the sense of a fresh mouth and in tooth discoloration control.^55^, ^56^ In August 1960, the American Dental Association (ADA) for the first time recognized a dentifrice with fluoride to have therapeutic value in fighting tooth decay.^57^ Since fluoride dentifrices first became available, many formulation changes regarding fluoride type, concentration and abrasive systems have been made to improve stability, compatibility and bioavailability of active ingredients.^58^ Even chemical agents have been added for the improved treatment of bad breath, staining, caries, gingivitis, dental plaque, dental calculus, demineralization and dentinal hypersensitivity.^56^, ^59^ Because plaque control plays a paramount role in the aetiology of caries and periodontal disease 60 and plaque formation on teeth cannot be stopped, disturbing plaque accumulation is of major importance.^61^ The aim of the present review was to investigate whether dentifrice can play a role as plaque‐reducing agent. Nearly all the dentifrices in the included studies of this SR appeared to provide a significant inhibiting effect on plaque regrowth in comparison with rinsing with water or saline.

The 4‐day nonbrushing model design, developed by Addy et al,^15^ has been extensively used to investigate the effects of mouthrinses or dentifrice slurries. For the latter, the model utilizes an aqueous dentifrice slurry and examines the effects of such treatments on plaque regrowth over a 4‐day period of no oral hygiene following a dental prophylaxis. By comparison with controls, the relative biological effects of antimicrobial ingredients incorporated into dentifrices can be determined. This design approximates the dilatation of a dentifrice with saliva that occurs with normal use of such products.^18^, ^62^ This study design prevents the complicating effects of mechanical toothbrushing.^15^, ^18^, ^63^, ^64^ Consequently, the Hawthorne effect, the effect often cited as being responsible for oral health improvements of control groups that receive placebo treatments,^65^ may be absent or limited. One could question whether a slurry achieves the same antibacterial effect as that obtained by the original dentifrice. Addy et al^15^ attempted to produce dentifrice slurries of comparable concentration to that delivered by toothbrush. Therefore, 3 g/10 mL of each paste was employed, based on the normal quantity of toothpaste used on a brush was reported to be 1.45 g 62 which is diluted approximately 1 in 4 by saliva.^15^ Moran et al^66^ have pointed out that an antimicrobial product that is proved ineffective in such a study would also have no effect if used with a toothpaste and mechanical cleaning.^15^

The results of this SR agree with those of other studies which do include the mechanical action of toothbrushing. Experiments over a 24‐hour duration confirmed toothbrushing with dentifrice to form less plaque postbrushing compared with brushing with water alone.^10^, ^11^, ^67^, ^68^ Also, experiments ranging from four days to five weeks exhibited higher inhibition of plaque regrowth by brushing with dentifrices as opposed to that by brushing with water alone.^9^, ^12^, ^69^, ^70^, ^71^, ^72^

In the meta‐analyses of this SR, a high heterogeneity was demonstrated for the studies that evaluated the products according to the PI of Q&H Turesky et al^26^ and Plaque Area 15 indices. Since systematic reviews bring together studies that are diverse both clinically and methodologically, heterogeneity in their results is to be expected.^73^, ^74^, ^75^ The performed subanalysis on the reported dentifrice ingredients did not provide a clear explanation for differences between the experiments. The results could also be negatively influenced by using prophylaxis in all the studies. Because prophylaxis removes the acquired pellicle, the absence of a pellicle that serves as a reservoir could reduce the substantivity of some therapeutic ingredients.^13^ It is the question of the extent to which this has influenced the results of the included studies.

Another source of clinical heterogeneity is the rinsing protocols in the included studies. The rinsing time was one minute except for the 30‐second rinsing in the study by Owens et al^44^ It is conceivable that when the amount of plaque removal is highly dependent on the brushing time 76 this is also valid for the rinsing time.^77^ Conversely, Paraskevas et al^78^ observed that rinsing for 30s was sufficient for plaque‐covered surfaces to come into contact with the mouthwash, and similarly Van der Weijden et al^79^ found no significant difference in rinsing time whether the participants rinsed for 15, 30, 30 or 60s with 0.2% chlorhexidine in the level of plaque after 72 hours of nonbrushing. Because of the high unexplained heterogeneity, the effect sizes and accompanying confidence intervals should be interpreted with caution. Nevertheless, given the clear direction of nearly all the observed effects in favour of using dentifrice, it is reasonable to be confident in the results presented.

The meta‐analysis allowed for a subgroup analysis on the reported dentifrice ingredients some of which have claimed antiplaque activity. These were sodium fluoride (NaF), sodium monofluorophosphate (MFP), stannous fluoride (SnF), triclosan (Tcs) and baking soda. Irrespective of the Plaque Index used (Q&H Turesky et al,^26^ Greene and Vermillion,^24^ Plaque Area 15), the Tcs product numerically exhibited the highest inhibition of plaque regrowth. Interestingly, both NaF and MFP products, which contained no specific ingredients brought forward for their antimicrobial effect, exhibited, irrespective of the Plaque Index used in all the meta‐analysis (Appendices S4, S5, and S6), a significant effect on the regrowth of plaque. Evidently, dentifrices contain more ingredients which exhibit inhibition of plaque regrowth of which SLS is the most commonly used ingredient.

Besides difference in means (DiffM) and 95% confidence intervals, we calculated also 95% prediction intervals. The advantage of also using prediction intervals is that it is more informative. It reflects the variation in treatment effects over different settings, including what effect is to be expected in future patients, such as the patients that a clinician is interested to treat.^29^ The prediction intervals were all below zero and suggest that dentifrice will be beneficial when applied in at least 95% of the individual study settings, an important finding for clinical practice.^80^, ^81^

Most systematic reviews with meta‐analyses are underpowered.^82^, ^83^ Trial sequential analysis (TSA) is a cumulative random‐effects meta‐analysis method that estimates a “required information size” (ie, required meta‐analysis sample size) using the same framework as sample size calculation for an individual RCT, but additionally accounting for heterogeneity and multiple comparisons when new RCTs are added. Also, before the required information size is reached, TSA constructs monitoring boundaries to determine when an estimated effect is so convincingly large (or small) that the conclusions are unlikely to change with more evidence.^42^, ^82^, ^84^

The TSA of the Greene and Vermillion 24 index suggested statistical evidence for this meta‐analysis. The number of participants almost reaches the information size, and the cumulative *Z*‐curve does cross the monitoring boundary. The TSA of the Q&H by Turesky et al^26^ index showed that the evidence was moderate.^41^ The number of participants does reach nearly the information size, and the cumulative *Z*‐curve does cross the monitoring boundary. The TSA of the Plaque Area Index^15^ showed inconclusive evidence.^41^ The cumulative *Z*‐curve does not cross the monitoring boundary before reaching the information size. Despite the latter, there are no indications that the conclusion of this systematic review is based on too little power of the underlying meta‐analysis.

### 4.1 Post hoc sensitivity analysis

In a crossover trial, each participant serves as his/her own control. A correlation coefficient describes how similar different measurements on interventions are within a participant.

Since the results of crossover trials are generally similar to those of parallel‐arm trials,^85^ the results of the crossover trials included in this MA were treated as parallel‐arm trials. However, treatment‐period interaction and carry‐over effects of crossover trials may jeopardize the validity of such simple inferences. Nevertheless, the results of the sensitivity analysis of the crossover trials with correlation coefficients of 0.5, 0.25 and 0 were in agreement with the results of the MA.

Several limitations were identified for this review. The composition of the dentifrices in the included studies was often not clear due to insufficient reporting. The majority of the included studies became available two or more decades ago. The manner of reporting did not follow current standards, such as CONSORT 2010 and TIDieR 2014. This limitation is also reflected in the results of the risk of bias assessment. This systematic review reinforces the importance of correct and complete reporting and adherence to standards, particularly the new TIDieR checklist 86 regarding the description and replication of interventions.

Other limitations are described in detail in the Appendix S9.

In summary, plaque scores of the dentifrices slurries in the 4‐day nonbrushing models demonstrate a reduction in plaque formation. The question is whether this effect is noticeable under normal home‐use conditions. Small reductions in plaque regrowth may reduce gingivitis and caries to a certain extent. Based on the findings of the present review, it is recommended that, with respect to plaque regrowth inhibition, a dentifrice should be used during toothbrushing. Future research may focus more specifically on active ingredients of dentifrices with assumed impact on dental plaque regrowth in different study models or on other reasons for using a dentifrice. In the future, dentifrice manufactures may reinforce its role as a nurturing dental cream.

## 5 CONCLUSION

The results of this review demonstrate moderate‐quality evidence for a weak inhibitory effect on plaque regrowth in favour of the use of a dentifrice.

## 6 CLINICAL RELEVANCE

### 6.1 Scientific rationale for the study

Twice‐daily toothbrushing with a fluoride dentifrice is a universal recommendation for personal oral care. A recent review has indicated that dentifrice does not provide an additional effect to toothbrushing with respect to plaque removal. The plaque regrowth inhibitory property of dentifrices has not yet been systematically studied.

### 6.2 Principal findings

This review demonstrates that toothpaste contributes to a reduction in plaque regrowth following a professional prophylaxis.

### 6.3 Practical implications

Although dentifrice does not contribute to the mechanical plaque removing efficacy, the addition of dentifrice enhances the lasting effect of toothbrushing. Active plaque‐inhibiting ingredients support the daily use of a regular dentifrice.

## CONFLICT OF INTEREST AND SOURCE OF FUNDING STATEMENT

The authors declare that they have no conflict of interests.

This research received no specific grant from any funding agency in the public, commercial or not‐for‐profit sectors. For this study, no funding was accepted, except for support from the listed institutions.

Ethical approval was not required.

Van der Weijden, Slot and their research team at ACTA have previously received either external advisor fees, lecturer fees or research grants from toothbrush and dentifrice manufacturers. Those manufacturers included Colgate, Dentaid, GABA, Lactona, Oral‐B, Procter & Gamble, Sara Lee, Sunstar and Unilever.
