# Supplementary material for: Is plaque regrowth inhibited by dentifrice? A systematic review and meta‐analysis with trial sequential analysis
Source: Int J Dent Hyg. 2018 Sep 27;17(1):27–38. doi: 10.1111/idh.12364 (PMC7379558; doi:10.1111/idh.12364)
Supplement: Supplementary file 1 [file IDH-17-27-s001.docx]

**Is plaque regrowth inhibited by dentifrice?**

*-A Systematic Review-*

***Version: 15-05-2018***

**C. Valkenburg**

**Fridus A. van der Weijden**

**Dagmar E. Slot**

**Online Supplementary Information**

**Online Supporting Information Legends**

**ONLINE Appendix S1.**

Overview of the studies processed for data extraction.

**ONLINE Appendix S2.**

Methodological quality and potential risk of bias scores of the individual included studies.

**ONLINE Appendix S3.**

End outcomes presented by and relative to plaque index and plaque area score.

**ONLINE Appendix S4a.**

Forest plot of END plaque scores using the Turesky *et al.* (26) modification of the Quigley & Hein (25) Plaque Index for the rinsing experiments using a dentifrice slurry or water/saline.

**ONLINE Appendix S5a.**

Forest plot of END plaque scores using the Greene & Vermillion (24) Index for the rinsing experiments using a dentifrice slurry or water/saline.

**ONLINE Appendix S6a.**

Forest plot of END plaque scores using the Addy *et al.* (15) modification of the Shaw & Murray (27) Stain Index for the rinsing experiments using a dentifrice slurry or water/saline.

**ONLINE Appendix S4b.**

Formal testing of publication bias in support of the meta-analysis presented in S8 for the rinsing experiments using the Turesky *et al.* (26) modification of the Q&H (25) Plaque Index.

**ONLINE Appendix S5b.**

Formal testing of publication bias in support of the meta-analysis presented in S9 for the rinsing experiments using the Greene & Vermillion (24) Plaque Index .

**ONLINE Appendix S6b.**

Formal testing of publication bias in support of the meta-analysis presented in S10 for the rinsing experiments using the Addy *et al.* (15) modification of the Shaw & Murray (27) Stain Index.

**ONLINE Appendix S7.**

Trial Sequential Analysis.

**ONLINE Appendix S8.**

Post-hoc sensitivity analysis of the cross-over trials included in this MA.

**ONLINE Appendix S9.**

Limitations related to the evidence that emerges from this review.

**ONLINE Appendix S1.**

Overview of the studies processed for data extraction.

| **Authors**  **(year)** | **Study design, duration** | **# Participants baseline (end),**  **gender,**  **age (mean/range),**  **oral prophylaxis (OP)** | **Groups**  ***Brands*** | **Regimen:**  **use &**  **instructions** | **Conclusions of the original authors** |
| --- | --- | --- | --- | --- | --- |
| **Arweiler et al. (2002)** (17) | RCT  Cross-over (w.o. 10 days) | 8(8)  Employees and dental students  Germany  ♀: 4 (50%◊)  ♂: 4 (50%◊)  Mean age: 26.8 (1.8)  Age range: ≥18  OP: yes | **•** **Tcs DFS** (Colgate Total ®, Colgate-Palmolive Hamburg, Germany)  **•** **BS DFS** (Parodontax mit Fluorid ®, Ratingen, Germany)  **• Water** | Twice daily rinsing (morning and evening) for 1 min;  3g dentifrice /10 ml water  Supervision: only first time | Tcs DFS has shown a significant action on plaque regrowth.  BS DFS revealed a more moderate but still significant effect. |
| **Binney**  **et al. (1997)** (45) | RCT  Cross-over (w.o.9 days) | 12 (11)  Population Dental School, UK  ♀: 3  ♂: 9  Mean age: *22*  Age range: 21-26  OP: yes | **•** **Tcs-co DFS**; triclosan (0.3%) copolymer, sodium fluoride, sodium monofluorophosphate, Colgate Total®, Colgate-Palmolive, Surrey, UK  **• Tcs-Zn DFS**; triclosan (0.3%) zinc citrate, sodium fluoride, Mentadent P®, Unilever, Port Sunlight Laboratories, Merseyside, England  **• SnF DFS**; stannous fluoride (0.454%) ,  Crest Gum Care®, Procter and Gamble, Middlesex, UK  **• Water** | Twice daily rinsing (± 9 a.m. and ± 5 p.m.) for 1 min;  3g dentifrice /10 ml water  Supervision: yes | All DFS were more effective than water but there were no differences between the active treatments. |
| **Owens**  **et al. (1997)** (44) | RCT  Cross-over (w.o.10 days) | 29 (20)  Population UK?  ♀: 10  ♂: 10  Mean age: 23,5◊  Age range: 21-38◊  OP: yes | **• MFP+NaF DFS (**sodium fluoride, sodium monofluorophosphate, Colgate®, Colgate-Palmolive, Guildford, UK)  **• Water** | Twice daily rinsing (8.30-9.30 a.m. and 4.00-5.00 p.m.) for 30 seconds;  3g dentifrice /10 ml water  Supervision: yes | Dentifrice slurry rinses resulted in less plaque than water, differences which were not significant. |
| **Binney**  **et al. (1996)** (48) | RCT  Cross-over (w.o.3 days) | 18 (17)  Population of employees and students Dental School, UK  ♀: 9  ♂: 9  Mean age: *nr*  Age range: 18-34  OP: yes | **• Tcs DFS** (sodium fluoride, triclosan (0.3%) and sodium monofluorophosphate, Colgate Total®)  **•** **NaF DFS** (sodium fluoride (0.32%) Crest Regular®)  **•** **NaF- pyro DFS** (pyrophosphates (0.5%), sodium fluoride (0.24%) Crest Tartar Control®)  **•** **MFP+NaF DFS** (sodium fluoride and sodium monofluorophosphate (0.76%) Colgate Regular®)  **• MFP DFS** (sodium monofluorophosphate (0.3%) Colgate Gel®)  **• Water** | Twice daily rinsing for 1 min;  3g dentifrice /10 ml water  Supervision: yes | The tested dentifrices demonstrated a significantly greater plaque inhibition than that of water when used as a rinse as the only form of oral hygiene. |
| **Binney**  **et al. (1995)** (47) | RCT  Cross-over (w.o.3 days) | 20 (18)  Population of employees and students Dental School, UK  ♀: 15  ♂: 5  Mean age: *nr*  Age range: 18-31  OP: yes | **• NaF DFS** (NaF and monofluorophosphate, Colgate Regular®, Colgate- Palmolive Company, Piscataway, New Jersey, USA)  **• Tcs DFS** (triclosan (0.3%) copolymer, Colgate Gum Protection Formula®, Colgate- Palmolive Company, Piscataway, New Jersey, USA)  **•** **Saline** (sodium chloride (0.9%)) | Twice daily rinsing for 1 min;  3g dentifrice /10 ml tap water  Supervision: yes | Dentifrice preparations were significantly better than the saline rinse. |
| **Binney**  **et al. (1992)** (16) | RCT  Cross-over (w.o.3 days) | 18 (18)  Population UK?  ♀:10  ♂: 8  Mean age: *nr*  Age range: 20-29  OP: yes | **• MFP+NaF DFS** (sodium monofluorophosphate (0.76%w/w), sodium fluoride (0.1%w/w) Colgate- Palmolive, Guildford, UK)  **• Saline** (sodium chloride (0.9%w/v), Steripak Ltd, Runcorn, UK) | Twice daily rinsing for 1 min;  3g dentifrice /10 ml water  Supervision: no | There were no significant differences between saline or a dentifrice slurry. |
| **Addy et al. (1990)** (46) | RCT  Cross-over (w.o.2-3 days) | 15 (15)  Population of staff and students Dental School, UK  ♀: 9  ♂: 6  Mean age: *nr*  Age range: 20-27  OP: yes | **•** **NaF DFS** (sodium fluoride (1.5%) SLS†)  **• Tcs DFS** (sodium fluoride, 0.3% triclosan (2.5%) SLS†)  **• Tcs-co DFS** (sodium fluoride, 2% PVM/MA, 0.3% triclosan (2.5%) SLS†)  **• SnF DFS** (non-commercial stannous fluoride (0.4%), (1.2%) SLS†)  **• SnF-SnCl DFS** (non-commercial stannous fluoride (0.4%), stannous chloride (0.24%), (1.2%) SLS†)  **• SnF gel DFS** (stannous fluoride gel (0.4%), Schere Gel, Kam)  **• Saline** (Isotonic saline, Dental School Pharmacy, Cardiff, UK)  ^†^ Colgate-Palmolive Company, Research and Development Division, Piscataway, New Jersey, USA | Twice daily rinsing for 1 min;  3g dentifrice /10 ml water  Supervision: ? | Commercially available dentifrices have plaque inhibitory effects which so far appear difficult to improve upon by the addition of specific ingredients, in this case stannous fluoride or triclosan. |
| **Addy et al. (1983)** (15) | RCT  Cross-over (w.o. 3 days) | 10 (10)  Population employed Dental School, UK  ♀: 4  ♂: 6  Mean age: 27.6  Age range: 22-39  OP: yes | **• MFP DFS** (monofluorophosphate (0.76%))  **• MFP+NaF DFS** (monofluorophosphate (0.76%), sodium fluoride (0.1%))  **• MFP-Zn DFS** (monofluorophosphate (0.8%), zinc citrate (0.5%))  **• NaF DFS** (sodium fluoride (0.24%))  **• SnF DFS** (stannous fluoride (0.4%))  **• Water** | Twice daily at 10 a.m. and 10 p.m. rinsing for 1 min;  3g dentifrice /10 ml water  Supervision: partly | At 96 h plaque areas were significantly less with dentifrice rinses compared with water. |

BS: baking soda; Parodontax with fluoride;

co: copolymer

DFS: dentifrice slurry

*nr*.: not reported/unknown

OP: at the initial appointment, all teeth were thoroughly scaled and polished

MFP: sodium monofluorophosphate

NaF: sodium fluoride

Pyro: pyrophosphate

SLS: sodium lauryl sulfate

SnCl: stannous chloride

SnF: stannous fluoride-containing dentifrice

Tcs: triclosan

w.o.: washout period for a crossover design

Zn: zinc citrat

**ONLINE Appendix S2.**

Methodological quality and potential risk of bias scores of the individual included studies.

|  | | Arweiler et al. (2002) (17) | Binney et al. (1997) (45) | Owens et al. (1997) (44) | Binney et al. (1996) (48) | Binney et al. (1995) (47) | Binney et al. (1992) (16) | Addy et al. (1990) (46) | Addy et al. (1983) (15) |
| --- | --- | --- | --- | --- | --- | --- | --- | --- | --- |
|  | Study design | cross over | cross over | cross over | cross over | cross  over | cross  over | cross  over | cross  over |
| **Internal validity** | Random allocation* | + | + | + | + | + | + | + | + |
|  | Allocation concealment | *nr* | *nr* | *nr* | *nr* | *nr* | *nr* | *nr* | *nr* |
|  | Blinded to product* | na | na | na | na | na | na | na | na |
|  | Blinded to examiner* | **+** | **+** | **+** | **+** | **+** | **+** | **+** | **+** |
|  | Blinding during statistical analysis | *nr* | *nr* | *nr* | *nr* | *nr* | *nr* | *nr* | *nr* |
|  | Balanced experimental groups* | **+** | **+** | **+** | **+** | **+** | **+** | **+** | **+** |
|  | Reported loss to follow-up* | + | + | + | + | + | + | + | + |
|  | # (%) of drop-outs | 0 (0%)◊ | 1 (8%)◊ | 9 (31%)◊ | *1 (6%)◊* | 2 (10%)◊ | 0 (0%)◊ | 0 (0%)◊ | 0 (0%)◊ |
|  | Treatment identical, except for intervention* | **+** | **+** | **+** | **+** | **+** | **+** | **+** | **+** |
| **External validity** | Representative population group | + | **+** | **+** | **+** | **+** | + | + | + |
|  | Eligibility criteria defined* | + | + | + | + | + | + | + | + |
|  | Sample size calculation and power | *nr* | **+** | **+** | *nr* | *nr* | *nr* | *nr* | *nr* |
|  | Point estimates presented for the primary outcome | **+** | **+** | **-** | **+** | **+** | **+** | **+** | **+** |
|  | Measures of variability presented for the primary outcome | **+** | **+** | **-** | **+** | **+** | **+** | **+** | **+** |
|  | Unit of analysis | **Subject** | **Subject** | **Subject** | **Subject** | **Subject** | **Subject** | **Subject** | **Subject** |
|  | Included a per protocol analysis | **+** | **+** | **+** | *+* | **+** | **+** | *+* | **+** |
|  | Included an intention-to-treat analysis | **?** | **+** | **+** | **?** | **?** | **?** | **?** | **?** |
| **Clinical aspects** | Validated measurement | **+** | **+** | **+** | **+** | **+** | **+** | **+** | **+** |
|  | Calibration examiner | **+** | *nr* | **+** | **+** | + | *nr* | *nr* | *nr* |
|  | Reproducibility data shown | **-** | **-** | **-** | **-** | **-** | **-** | **-** | **-** |
| Authors’ estimated risk of bias | | **low** | **low** | **low** | **low** | **low** | **low** | **low** | **low** |

Each aspect of the score list was given a rating of ‘+’ for an informative description of the item at hand and a study design meeting the quality standard,

‘-’ for an informative description without a study design that met the quality standard and ‘?’ for missing or insufficient information. When random allocation, defined eligibility criteria, blinding of examiners and patients, balanced experimental groups, identical treatment between groups (except for intervention) and report of follow-up were present, the study was classified as having a low risk of bias. When one of these seven criteria was missing, the study was considered to have a moderate potential risk of bias. When two or more of these criteria were missing, the study was considered to have a high potential risk of bias, as proposed by Van der Weijden et al. (2009).

na: not applicable

*nr*: not reported/unclear

+: yes

-: no

*: reporting criteria for estimating the potential risk of bias

◊: calculated by the authors of this review based on the data presented in the selected paper

**Online Appendix S3.** End outcomes presented by and relative to plaque index and plaque area score.

1. End mean (SD) for the plaque index according the Turesky *et al.* (26) modification of the Quigley & Hein (25) Plaque Index. Presented per included study by the group of interests for this review.

| **Study** | **Group** | **END mean (sd)** | **N** |
| --- | --- | --- | --- |
| Arweiler et al. (2002) (17) | Tcs DFS | 1.85 (0.35) | 8 |
|  | BS DFS | 2.59 (0.37) | 8 |
|  | Water | 2.96 (0.49) | 8 |
| Binney et al. (1992) (16) | MFP+NaF DFS | 2.3 (0.23) | 18 |
|  | Saline | 2.28 (0.33) | 18 |
| Binney et al. (1995) (47) | NaF DFS | 2.35 (0.056) | 18 |
|  | Tcs DFS | 2.21 (0.058) | 18 |
|  | Saline | 2.66 (0.065) | 18 |
| Binney et al. (1996) (48) | MFP DFS | 2.49 (0.140◊) | 17 |
|  | MFP+NaF DFS | 2.46 (0.144◊) | 17 |
|  | NaF DFS | 2.39 (0.140◊) | 17 |
|  | NaF-pyro DFS | 2.4 (0.140◊) | 17 |
|  | Tcs DFS | 2.24 (0.144◊) | 17 |
|  | Water | 2.65 (0.140◊) | 17 |
| Binney et al. (1997) (45) | SnF DFS | 2.14 (0.40) | 12 |
|  | Tcs-co DFS | 2.14 (0.483) | 11 |
|  | Tcs-Zn DFS | 2.26 (0.286) | 11 |
|  | Water | 2.49 (0.328) | 11 |
| Owens et al. (1997) (44) | MFP+NaF DFS | ?? (??) | 20 |
|  | Water | ?? (??) | 20 |

1. End mean (SD) for the plaque index according the Greene & Vermillion Plaque Index (1960). Presented per included study by the group of interests for this review.

| **Study** | **Group** | **END mean (sd)** | **N** |
| --- | --- | --- | --- |
| Addy et al. (1983) (15) | MFP DFS | 0.995 ◊ (0.255◊) | 10 |
|  | MFP+NaF DFS | 1.03 ◊ (0.17◊) | 10 |
|  | MFP-Zn DFS | 0.96 ◊ (0.135◊) | 10 |
|  | NaF DFS | 1.02 ◊ (0.165◊) | 10 |
|  | SnF DFS | 0.995 ◊ (0.14◊) | 10 |
|  | Water | 1.23 ◊ (0.26◊) | 10 |
| Addy et al. (1990) (46) | NaF DFS | 2.22 (0.4) | 15 |
|  | SnF DFS | 2.22 (0.51) | 15 |
|  | SnF gel DFS | 2.27 (0.42) | 15 |
|  | SnF-SnCl DFS | 2.26 (0.39) | 15 |
|  | Tcs DFS | 2.23 (0.49) | 15 |
|  | Tcs-co DFS | 2.26 (0.46) | 15 |
|  | Saline | 2.55 (0.54) | 15 |

1. End mean (SD) for the Plaque Area Index. Presented per included study by the group of interests for this review.

| **Study** | **Group** | **END mean (sd)** | **N** |
| --- | --- | --- | --- |
| Arweiler et al. (2002) * (17) | Tcs DFS | 13.4% (9.9%) | 8 |
|  | BS DFS | 19.2% (9.5%) | 8 |
|  | Water | 27.4% (14.7%) | 8 |
| Binney et al. (1996) ** (48) | MFP DFS | 1.28 (0.210◊) | 17 |
|  | MFP+NaF DFS | 1.15 (0.219◊) | 17 |
|  | NaF DFS | 1.1 (0.210◊) | 17 |
|  | NaF-pyro DFS | 1.12 (0.210◊) | 17 |
|  | Tcs DFS | 0.97 (0.219◊) | 17 |
|  | Water | 1.47 (0.210◊) | 17 |
| Binney et al. (1995) ** (47) | NaF DFS | 0.99 (0.056) | 18 |
|  | Tcs DFS | 0.87 (0.062) | 18 |
|  | Saline | 1.34 (0.069) | 18 |
| Binney et al. (1992) ** (16) | MFP+NaF DFS | 2.26 (1.06) | 18 |
|  | Saline | 2.26 (0.98) | 18 |
| Addy et al. (1990) ** (46) | NaF DFS | 1.15 ◊ (1.07) ◊ | 15 |
|  | SnF DFS | 1.15 ◊ (1.07) ◊ | 15 |
|  | SnF gel DFS | 1.16 ◊ (1.07) ◊ | 15 |
|  | SnF-SnCl DFS | 1.16 ◊ (1.07) ◊ | 15 |
|  | Tcs DFS | 1.16 ◊ (1.08) ◊ | 15 |
|  | Tcs-co DFS | 1.16 ◊ (1.09) ◊ | 15 |
|  | Saline | 1.25 ◊ (1.12) ◊ | 15 |
| Addy et al. (1983) ** (15) | MFP DFS | 0.895 $◊$ (0.485◊) | 10 |
|  | MFP+NaF DFS | 1.138 ◊ (0.393◊) | 10 |
|  | MFP-Zn DFS | 0.833 ◊ (0.249◊) | 10 |
|  | NaF DFS | 0.933 ◊ (0.457◊) | 10 |
|  | SnF DFS | 0.958 ◊ (0.370◊) | 10 |
|  | Water | 0.998 ◊ (0.469◊) | 10 |

* plaque area index previously described:

Arweiler, N. B., Donos, N., Netuschil, L., Reich, E. & Sculean, A. (2000) Clinical and antibacterial effect of tea tree oil--a pilot study. Clinical Oral Investigations 4, 70-73.

Arweiler, N. B., Netuschil, L. & Reich, E. (2001) Alcohol-free mouthrinse solutions to reduce supragingival plaque regrowth and vitality. A controlled clinical study. Journal of Clinical Periodontology 28, 168-174.

** plaque area index previously described:

Addy, M., Willis, L. & Moran, J. (1983) Effect of toothpaste rinses compared with chlorhexidine on plaque formation during a 4-day period. *Journal of Clinical Periodontology* **10,** 89-99.

◊: calculated by the authors of this review based on the data presented in the selected paper

♦: additional data provided by the original authors

BS: baking soda; Parodontax with fluoride

DFS: dentifrice slurry

co: copolymer

MFP: sodium monofluorophosphate

NaF: sodium fluoride

Pyro: pyrophosphate

SLS: sodium lauryl sulfate

SnCl: stannous chloride

SnF: stannous fluoride-containing dentifrice

Tcs: triclosan

Zn: zinc citrate

**ONLINE Appendix S4a.**

Forest plot of END plaque scores using the Turesky *et al.* (26) modification of the Q&H (25) Plaque Index for the rinsing experiments using a dentifrice slurry or water/saline.

A chi-square test resulting in a p value < 0.1 was considered to be an indication of significant statistical heterogeneity. As an approximate guide for assessing the magnitude of inconsistency across studies, an I2 statistic of 0–40% was interpreted as potentially not important, and for a statistic above 40%, moderate to considerable heterogeneity (19).

See for the funnel plot based on this forest plot Online Appendix S4b.

**ONLINE Appendix S5a.**

Forest plot of END plaque scores using the Greene & Vermillion (24) Index for the rinsing experiments using a dentifrice slurry or water/saline.

A chi-square test resulting in a p value < 0.1 was considered to be an indication of significant statistical heterogeneity. As an approximate guide for assessing the magnitude of inconsistency across studies, an I2 statistic of 0–40% was interpreted as potentially not important, and for a statistic above 40%, moderate to considerable heterogeneity (19).

See for the funnel plot based on this forest plot Online Appendix S5b.

**ONLINE Appendix S6a**.

Forest plot of END plaque scores using the Addy *et al.* (15) modification of the Shaw & Murray (27) Stain Index for the rinsing experiments using a dentifrice slurry or water/saline.

A chi-square test resulting in a p value < 0.1 was considered to be an indication of significant statistical heterogeneity. As an approximate guide for assessing the magnitude of inconsistency across studies, an I2 statistic of 0–40% was interpreted as potentially not important, and for a statistic above 40%, moderate to considerable heterogeneity (19).

See for the funnel plot based on this forest plot Online Appendix S6b.

**ONLINE Appendix S4b.**

Formal testing of publication bias in support of the meta-analysis presented in S8 for the rinsing experiments using the Turesky et al. (26) modification of the Q&H (25) Plaque Index. Funnel plot belongs to forest plot Online Appendix S4a.

Egger’s test shows a non-significant *p*-value (*p* = 0.4431).

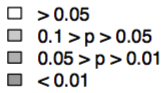


The above funnel is centered at the model estimate. Color of line represent random effects estimate. The filled circles represent estimated treatment effect (mean difference) and its precision (standard error) for each individual experiment. The open circles are missing studies filled in by a Trim-and-Filled Method. The basic idea of the trim-and-fill method is to add studies to the funnel plot until it becomes symmetric (34). The Counter-Enhanced Funnel plot is an enhancement to the usual funnel plot proposed to allow considering the statistical significance of study estimates (49). Contour lines representing well established levels of statistical significance are added to the funnel plot to indicate regions where a test of treatment effect is significant. Contour lines indicating conventional milestones in levels of statistical significance (e.g., <0.01, <0.05, <0.1) are added (34). P-values correspond to a trial’s treatment effect (51). The unshaded (i.e., white) region in the middle corresponds to non-significant results, the medium gray region to significant results at the 5% level and the dark gray region to the 1% level. The counter-funnel plot could be used naturally in conjunction with the trim-and-fill method because the latter informs the likely location of missing studies (49).

Visually, shows the funnel plot asymmetry. However, Egger’s test for asymmetry is not significant (*p*=0.443), indicating a publication bias mechanism is not a major cause for concern here (50).

**ONLINE Appendix S5b.**

Formal testing of publication bias in support of the meta-analysis presented in S9 for the rinsing experiments using the Greene & Vermillion (24) Plaque Index. The funnel plot indicates that publication bias is not likely. Funnel plot belongs to forest plot Online Appendix S5a. Egger’s test shows a significant *p*-value (*p* = 0.004).

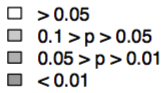


Visually, the plot shows asymmetry. Missing studies probably lie within the white non-significant white region (50).

Egger’s test shows a significant *p*-value (*p*= 0.004) leading to rejection of the null hypothesis of symmetry in the funnel plot (34).

**ONLINE Appendix S6b.**

Formal testing of publication bias in support of the meta-analysis presented in S10 for the rinsing experiments using the Addy *et al.* (15) modification of the Shaw and Murray (27) Stain Index. The funnel plot indicates that publication bias is not likely. Funnel plot belongs to forest plot Online Appendix S6.

Egger’s test shows a significant *p*-value (*p*= 0.004).

Visually, the plot shows asymmetry. Missing studies probably lie in the significant colored and shaded region (50). Egger’s test shows a significant *p*-value (*p*= 0.004) leading to rejection of the null hypothesis of symmetry in the funnel plot (34).

**ONLINE** **Appendix S7.**

**Trial Sequential Analysis.**

**Appendix S7-1)** TSA of the END plaque scores using the Turesky *et al.* (26) modification of the Q&H (25) Plaque Index for the rinsing experiments using a dentifrice slurry or water/saline.

TSA shows that the evidence is moderate. The number of participants does reach nearly the information size and the cumulative Z-curve does cross the monitoring boundary.

The cumulative blue Z-curves were constructed with each cumulative Z-value calculated after including a new trial according to publication date. Crossing of the two-sided Z = 1.96 provides a traditionally significant result. Crossing of the red trial sequential monitoring boundaries is needed to obtain reliable evidence adjusted for random error risk. Z-curves not crossing Z= 1.96 indicate absence of evidence if the information size is not reached or lack of the predefined intervention effect if the information size is not reached (83). The green dotted lines represent the traditional boundary. The vertical red line represents the estimated heterogeneity-adjusted required information size, the number of participants for the meta-analysis sample size.

**Appendix S7-2)** TSA of the END plaque scores using the Greene & Vermillion (24) Index for the rinsing experiments using a dentifrice slurry or water/saline.

TSA suggests that the statistical evidence is firm for this meta-analysis. The number of participants does reach the information size and the cumulative Z-curve does cross the monitoring boundary.

The cumulative blue Z-curves were constructed with each cumulative Z-value calculated after including a new trial according to publication date. Crossing of the two-sided Z = 1.96 provides a traditionally significant result. Crossing of the red trial sequential monitoring boundaries is needed to obtain reliable evidence adjusted for random error risk. Z-curves not crossing Z= 1.96 indicate absence of evidence if the information size is not reached or lack of the predefined intervention effect if the information size is not reached (83). The green dotted lines represent the traditional boundary. The vertical red line represents the estimated heterogeneity-adjusted required information size, the number of participants for the meta-analysis sample size.

**Appendix S7-3)** TSA of the END plaque scores using the Addy *et al.* (15) modification of the Shaw & Murray (27) Stain Index for the rinsing experiments using a dentifrice slurry or water/saline.

TSA shows inconclusive evidence. The cumulative Z-curve does not cross the monitoring boundary before reaching the information size.

The cumulative blue Z-curves were constructed with each cumulative Z-value calculated after including a new trial according to publication date. Crossing of the two-sided Z = 1.96 provides a traditionally significant result. Crossing of the red trial sequential monitoring boundaries is needed to obtain reliable evidence adjusted for random error risk. Z-curves not crossing Z= 1.96 indicate absence of evidence if the information size is not reached or lack of the predefined intervention effect if the information size is not reached (83). The green dotted lines represent the traditional boundary. The vertical red line represents the estimated heterogeneity-adjusted required information size, the number of participants for the meta-analysis sample size.

**ONLINE Appendix S8.**

Post-hoc sensitivity analysis of the cross-over trials included in this MA.

**Appendix S8-1)** Post-hoc sensitivity analysis of the END plaque scores using the Turesky *et al.* (26) modification of the Q&H (25) Plaque Index for the rinsing experiments using a dentifrice slurry or water/saline. Sensitivity analysis with correlation values of 0, 0.25 and 0.5.

**Appendix S8-2)** Post-hoc sensitivity analysis of the END plaque scores using the Greene & Vermillion (24) Index for the rinsing experiments using a dentifrice slurry or water/saline. Sensitivity analysis with correlation values of 0, 0.25 and 0.5.

**Appendix S8-3)** Post-hoc sensitivity analysis of the END plaque scores using the Addy *et al.* (15) modification of the Shaw & Murray (27) Stain Index for the rinsing experiments using a dentifrice slurry or water/saline. Sensitivity analysis with correlation values of 0, 0.25 and 0.5.

**ONLINE Appendix S9.**

**Limitations related to the evidence that emerges from this review.**

Limitations.

- The included studies range in publication date from 1983 to 2002. The many years since publication precluded trying to contact authors for further information, and this is reflected in the studies included in the review. Five out of eight included studies in this review were published before the first publication of the CONSORT Statement in 1996. The quality of conduct and reporting of RCTs has improved greatly after that time.

- In the current study, the exact composition of the used dentifrices, which is no exception in other studies that evaluate dentifrices, is not clear. Therefore, when evaluating dentifrice studies, it is often unclear which components present or absent in dentifrices such as detergents with their known inhibitory activity (46, 64), may have been responsible for the results found.

- It is also conceivable that the results of the inhibition of regrowth by dentifrices could be more extensive in a population with higher mean plaque scores. The participants in the included studies were mainly dental students, dental staff or employees of a dental school. It may be assumed that their oral health and attitudes are of a high level. One can imagine that in a less dentally mindful population with possibly more average plaque scores, the effects of the inhibition of regrowth by using dentifrices would be more extensive.

- Blinding of dentifrice slurry or water is a problem.

- Slurry is not water. The effect of a slurry itself on water is unknown.

- The compliance of the given protocols may be considered as an important factor in the study outcomes. Four out of eight studies were under supervision (44, 45, 47, 48). One study was not under supervision but returns of each product suggested good compliance by all participants (16). One study was partly under supervision (15) and one only the first time (17). Only for one study supervision was unknown (46).

- The cross-over design was considered appropriate to include in this SR. Neither carry-over nor period effects were reported as to be a problem. However, data of the individual participants from the included studies was not available for this SR. By ignoring the cross-over design and to treat as different parallel groups, the data could be included in a meta-analysis. This approach ignores the fact that the same patients appear in both arms of the study and so they are not independent of each other, as required in the standard statistical methods. At best, it is conservative as it ignores the within-patient correlation (52). So, studies would be rather under-weighted than over-weighted.

- There has been much criticism of the use of plaque indices, relating particular to their resolution. For example, if one were using the Quigley Hein index, it would be possible for a tooth’s plaque level to decrease by 50% and yet would still be scored the same (74). Other difﬁculties lie with the subjective nature of the indices and the need for examiner training, often increasing the cost of clinical trials, as does the need for a clinician to conduct the examination (74).

- The dose of dentifrice in the slurry method is increased just greater than twofold compared to that normally delivered on a toothbrush but approximates the same concentration of dentifrice used on a brush after salivary dilution (62). What influence this increased dose has on plaque accumulation is unknown, but probably minimal (45).

- It may be inaccurate to compare the results of the tests of statistical heterogeneity between factor subgroups because the subgroups may not have sufficient statistical power with respect to the numbers of trials and participants to detect heterogeneity (73).
